# Prevalence of hyperuricemia and its associations with hyperinsulinemia and hepatic injury among adolescents with obesity in Guangxi, China

**DOI:** 10.3389/fnut.2026.1864474

**Published:** 2026-08-28

**Authors:** Binlin Chen, Xiuhua Pan, Rui Jiang, Xiandan Zhang, Fuli Ya, Jing Huang

**Affiliations:** 1 Department of Clinical Nutrition, Maternal and Child Health Hospital of Guangxi Zhuang Autonomous Region, Nanning, China; 2 Children and Adolescents Weight Management Center, Maternal and Child Health Hospital of Guangxi Zhuang Autonomous Region, Nanning China; 3 Department of Clinical Nutrition, The People’s Hospital of Guangxi Zhuang Autonomous Region, Nanning, China; 4 Department of Nutrition, School of Public Health, Dali University, Dali, Yunnan, China; 5 Department of Obstetrics, Maternal and Child Health Hospital of Guangxi Zhuang Autonomous Region, Nanning, China

**Keywords:** adolescent, alanine aminotransferase, China, hyperinsulinemia, hyperuricemia, metabolic risk factors, obesity

## Abstract

**Objective:**

To investigate the prevalence of hyperuricemia (HUA) in adolescents with obesity in Guangxi, southern China, and explore its associations with hyperinsulinemia and hepatic injury.

**Methods:**

A total of 200 adolescents with obesity aged 10–18 years who visited the Children and Adolescents Weight Management Center of Maternal and Child Health Hospital of Guangxi Zhuang Autonomous Region from May to December 2025 were enrolled. According to the *Clinical Practice Consensus Statement 2025: Management of Hyperuricemia and Gout in Adolescents* (fasting serum uric acid ≥ 420 μmol/L for HUA), the subjects were divided into non-HUA and HUA groups. Anthropometric and fasting laboratory indicators were collected. Univariate analyses, multivariate logistic regression, and unadjusted restricted cubic spline (RCS) models were applied for statistical analyses.

**Results:**

The prevalence of HUA was 44.50% (89/200), with 47.59% (69/145) in males and 36.36% (20/55) in females. Univariate analysis showed significant between-group differences in age, body mass index z-score (BMIZ), low-density lipoprotein cholesterol (LDL-C), fasting insulin, alanine aminotransferase (ALT), dyslipidemia, and hyperinsulinemia (all *P* < 0.05). Multivariate logistic regression revealed that age > 12 years (OR = 2.60, 95%CI:1.19–5.68, *P* = 0.017), hyperinsulinemia (OR = 2.33, 95%CI:1.11–4.92, *P* = 0.026) and abnormal ALT (OR = 2.44, 95%CI:1.02–5.87, *P* = 0.046) were independent risk factors for HUA. Restricted cubic spline models revealed positive dose-response relationships between age, fasting insulin, ALT, BMIZ and the risk of HUA (all *P* for overall < 0.05).

**Conclusion:**

HUA is highly prevalent among adolescents with obesity in Guangxi. Older age, hyperinsulinemia, and elevated ALT are independently associated with HUA risk. These findings highlight the importance of comprehensive metabolic management, including insulin sensitivity and liver function assessment, in this high-risk population.

## 1 Introduction

The global epidemic of childhood and adolescent obesity has become one of the most pressing public health challenges of the 21st century (1, 2). In China, the prevalence of overweight and obesity among children and adolescents has surged dramatically over the past two decades. A national survey showed that the overweight rate of Chinese children and adolescents reached 20.36% and the obesity rate was 10.00% in 2020 (3). Furthermore, the World Obesity Federation World Obesity Atlas 2026 reported that the obesity prevalence among Chinese children aged 5–19-years reached 13.9% in 2025, substantially higher than the global average of 8.7% (4). Notably, significant regional disparities exist, with northern provinces generally exhibiting higher obesity rates than southern regions such as Guangxi; however, even these lower-prevalence areas have experienced rapid increases over the past decade, reflecting the nationwide expansion of the obesity epidemic (5).

Obesity during adolescence is particularly concerning as it is closely linked to a cluster of metabolic abnormalities that often persist into adulthood, significantly increasing the long-term risk of cardiovascular disease, type 2 diabetes, hypertension, and other chronic conditions (6). Among these metabolic complications, hyperuricemia (HUA), traditionally regarded as an adult-onset disorder, has emerged as a common issue in obese youth (7, 8). Elevated serum uric acid (SUA) levels are not only a precursor to gout but also an independent risk factor for hypertension, metabolic syndrome, chronic kidney disease, and cardiovascular events (9).

The newly published *Clinical Practice Consensus Statement 2025: Management of Hyperuricemia and Gout in Adolescents* provides a unified diagnostic criterion for adolescent HUA, defined as fasting serum uric acid ≥ 420 μmol/L for all adolescents aged 10–19 years, without gender-specific cut-offs (10). Serum uric acid levels are determined by the balance between hepatic purine production and urate elimination, with approximately 70 % of daily uric acid excreted via renal proximal tubule transporters (e.g., URAT1, GLUT9, ABCG2) and the remaining 30 % cleared through the intestinal tract. Disruption of this synthesis-excretion homeostasis, modulated by genetic variants, dietary fructose intake, low-grade inflammatory responses, gut microbiota dysbiosis, and adiposity-driven insulin resistance which up-regulates renal urate reabsorption transporters, contributes to the development of hyperuricemia in adolescents (11). A national pooled analysis of 11 population-based studies involving 54,580 Chinese children and adolescents aged 3–19 years revealed an overall HUA prevalence of 23.3 % (12), suggesting that HUA has become a significant public health problem in Chinese youth. However, previous studies have reported inconsistent prevalence rates of adolescent HUA, largely due to the lack of unified diagnostic criteria—especially the historical use of different standards for males and females (12). A recent regional study reported a 30.6 % prevalence of HUA among obese adults in Guangxi using the same SUA ≥ 420 μmol/L diagnostic threshold applied in our study (13), but data on the prevalence of HUA among adolescents with obesity in Guangxi remain limited, especially data generated within the past 5 years.

In response to the growing burden of obesity-related diseases, the Chinese government has launched the “Action Plan of Five-Health Promotion for Children and Adolescents (2026–2030),” mandating the integration of weight management services into healthcare systems and school health programs. In line with these national directives, many hospitals have established weight management centers, including our own Children and Adolescents Weight Management Center, to provide specialized care for this high-risk population.

Given the aforementioned research gaps and the context of national public health initiatives, this cross-sectional study aimed to: (1) determine the prevalence of HUA among adolescents with obesity aged 10–18 years in Guangxi, China, using the updated 2025 consensus criteria; (2) identify factors associated with HUA through comprehensive univariate and multivariate analyses; and (3) evaluate the independent associations of hyperinsulinemia and hepatic injury with HUA risk. We hypothesized that HUA would be highly prevalent in this population and would be independently associated with markers of insulin resistance and hepatic dysfunction. The findings are expected to provide valuable insights for clinical practice and public health interventions targeting HUA and its comorbidities in adolescents with obesity.

## 2 Materials and methods

### 2.1 Study subjects

A total of 200 adolescents with obesity aged 10–18 years were consecutively enrolled in this cross-sectional study among attendees of the Children and Adolescents Weight Management Center of Maternal and Child Health Hospital of Guangxi Zhuang Autonomous Region between May and December 2025. Sample size was calculated based on the standard formula for estimating population prevalence in cross-sectional research: *n* = Z^2^_α/2_ × P × (1-P)/d^2^. A two-sided α = 0.05 was adopted, and the acceptable absolute margin of error d was set at 0.07. We preset the anticipated hyperuricemia (HUA) prevalence *P* = 45 % for the following reasons: a local Guangxi survey of obese adults found a 30.6 % HUA rate, while domestic studies of obese adolescents reported HUA prevalence between 50.6 and 64.5 %; 45 % was selected as a conservative intermediate value to avoid biased sample size estimation. Calculation output showed a theoretical minimal sample size of 194 participants. To secure sufficient statistical power, we set the recruitment target to 200 eligible obese adolescents. A published regional cross-sectional study with a similar research theme enrolled 172 participants as a reference for preliminary sample planning (14). The diagnostic criteria for obesity were in accordance with the criteria set by the 2018 Chinese “Screening for Overweight and Obesity in School-Aged Children and Adolescents,” that is, BMI ≥ the 95th percentile of the same age and sex children and adolescents in China (15). The exclusion criteria were: (1) secondary obesity due to endocrine disorders (e.g., Cushing syndrome, hypothyroidism) or genetic syndromes; (2) adolescents taking drugs that affect uric acid metabolism, blood glucose, blood lipid and liver function in the recent 3 months; (3) adolescents with acute infection, fever and other acute diseases; (4) adolescents with incomplete key clinical data related to this study. According to the “*Clinical Practice Consensus Statement 2025: Management of Hyperuricemia and Gout in Adolescents*,” fasting serum uric acid ≥ 420 μmol/L was defined as HUA, and the subjects were divided into non-hyperuricemia group and hyperuricemia group accordingly (10). This study was approved by the Medical Ethics Committee of Maternal and Child Health Hospital of Guangxi Zhuang Autonomous Region [Approval No. (2025-7)1], and all the guardians of the subjects signed the informed consent form. Clinical trial number: not applicable.

### 2.2 Collection of general information and anthropometric indicators

All anthropometric indicators were measured by professional nurses with standardized methods, with each indicator measured three times and the average value adopted for analysis. Height was measured to the nearest 0.1 cm using a stadiometer with subjects standing barefoot in an upright position; body weight was measured to the nearest 0.1 kg using an electronic scale with subjects wearing light clothing and in a fasting state. Body mass index (BMI) was calculated as weight (kg) divided by the square of height (m^2^). BMI z-score (BMIZ) and height for age z-score (HAZ) were calculated in accordance with the World Health Organization (WHO) child growth standards for school-aged children and adolescents. Body fat percentage and visceral fat area were detected using a body composition analyzer (InBody S10, Biospace Co., Ltd., Seoul, Korea).

### 2.3 Collection of laboratory examination indicators

Venous blood samples were collected after an overnight fast of at least 10 h. Serum was separated by centrifugation and analyzed within 2 h of collection. Relevant indicators were measured by an automatic biochemical analyzer (Beckman AU5800, USA) with matched kits, including fasting insulin (FINS), fasting blood glucose (FBG), glycosylated hemoglobin (HbA1c), four blood lipid parameters (total cholesterol (TC), triglyceride (TG), high-density lipoprotein cholesterol (HDL-C), low-density lipoprotein cholesterol (LDL-C)), alanine aminotransferase (ALT) for liver function assessment and 25-hydroxyvitamin D [25(OH)D]). The diagnostic criteria for related metabolic abnormalities were as follows: Dyslipidemia was diagnosed as having one or more of the following: TC ≥ 5.17 mmol/L, TG ≥ 1.46 mmol/L, HDL-C < 1.03 mmol/L and LDL-C ≥ 3.36 mmol/L (16); hyperinsulinemia as the FINS level > 173 pmol/L, which was determined by our hospital’s laboratory reference range and supported by relevant literature (15); abnormal ALT was defined as serum ALT level > 40 U/L in this study (17); vitamin D deficiency was diagnosed as 25(OH)D < 20 ng/mL in accordance with guideline (18).

### 2.4 Statistical analysis

SPSS 25.0 statistical software and R 4.4.1 software were used for data analysis. The normality of continuous data was tested by the Shapiro-Wilk test. The measurement data conforming to normal distribution were expressed as mean ± standard deviation (Mean ± SD), and the comparison between groups was performed by independent sample *t*-test; the measurement data not conforming to normal distribution were presented as median (first quartile, third quartile) [M (Q1, Q3)], and the comparison between groups was performed by Mann-Whitney U test. The counting data were expressed as number (percentage) n (%), and the comparison between groups was performed by Chi-square test. Multivariate logistic regression analysis was used to screen the independent risk factors of HUA in adolescents with obesity. Restricted cubic spline (RCS) models were constructed in R to evaluate dose-response relationships between continuous predictors (age, fasting insulin, ALT, BMIZ) and the log odds of HUA. Three knots were placed at the 10th, 50th, and 90th percentiles of each predictor’s distribution. The median value (50th-percentile knot) of each variable was set as the reference point where odds ratio was fixed at 1. We reported P for overall association and P for nonlinearity to judge the significance of overall relationship and nonlinear components, respectively. The fitted curves with 95 % confidence intervals were visualized. A two-tailed *P*-value < 0.05 was considered statistically significant.

## 3 Results

### 3.1 Participant Characteristics, prevalence of hyperuricemia and univariate analysis

A total of 200 adolescents with obesity (145 males, 55 females) with median age 12.0 (IQR 11.0, 13.0) years were included in the study. The overall prevalence of HUA (SUA ≥ 420 μmol/L) was 44.50 % (89/200), with a prevalence of 47.59 % (69/145) in males and 36.36 % (20/55) in females.

The results of univariate analysis showed that there were significant differences in age, BMIZ, LDL-C, FINS, and ALT between the HUA group and the non-HUA group (*P* < 0.05), with the age (*Z* = −3.77, *P* < 0.001), BMIZ (*t* = −2.81, *P* = 0.005), LDL-C (*Z* = −2.05, *P* = 0.041), FINS (*Z* = −2.60, *P* = 0.009) and ALT (*Z* = −3.56, *P* < 0.001) in the HUA group being significantly higher than those in the non-HUA group. In terms of categorical data, the rates of dyslipidemia (χ^2^ = 9.01, *P* = 0.003), hyperinsulinemia (χ^2^ = 11.99, *P* < 0.001), abnormal ALT (χ^2^ = 15.04, *P* < 0.001), age > 12 years (χ^2^ = 12.32, *P* < 0.001) and BMIZ > 2.7 (χ^2^ = 4.18, *P* = 0.041) in the HUA group were significantly higher than those in the non-HUA group.

There were no significant differences in HAZ, body fat percentage, visceral fat area, gender, 25(OH)D level, TC, HDL-C, TG, FBG, HbA1c and the rate of vitamin D deficiency between the two groups (*P* > 0.05). Notably, the overall vitamin D deficiency rate in this cohort of adolescents with obesity was as high as 37.00 % (74/200), indicating a highly prevalent vitamin D deficiency in this population, though no significant difference in 25(OH)D levels (*Z* = −0.04, *P* = 0.969) or vitamin D deficiency rates (χ^2^ = 0.08, *P* = 0.784) was observed between the HUA group and non-HUA group. The detailed results are shown in Table 1.

**TABLE 1:** Univariate analysis of indicators between non-hyperuricemia group and hyperuricemia group.

Variables	Total (*n* = 200)	Non-HUA group (*n* = 111)	HUA group (*n* = 89)	Statistic	*P*
Age (y), M (Q_1_, Q_3_)	12.00 (11.00, 13.00)	11.00 (10.00, 12.00)	12.00 (11.00, 13.00)	*Z* = −3.77	**< 0.001**
Age, *n* (%)				χ^2^ = 12.32	**< 0.001**
≤ 12 y	138 (69.00)	88 (79.28)	50 (56.18)		
> 12 y	62 (31.00)	23 (20.72)	39 (43.82)		
Gender, *n* (%)				χ^2^ = 2.03	0.154
Male	145 (72.50)	76 (68.47)	69 (77.53)		
Female	55 (27.50)	35 (31.53)	20 (22.47)		
HAZ, Mean ± SD	0.92 ± 1.09	0.86 ± 0.97	0.99 ± 1.22	*t* = −0.81	0.419
BMIZ, Mean ± SD	2.72 ± 0.56	2.62 ± 0.55	2.84 ± 0.55	*t* = −2.81	**0.005**
BMIZ, *n* (%)				χ^2^ = 4.18	**0.041**
≤ 2.7	106 (53.00)	66 (59.46)	40 (44.94)		
> 2.7	94 (47.00)	45 (40.54)	49 (55.06)		
Body fat percentage, Mean ± SD	0.39 ± 0.06	0.39 ± 0.07	0.38 ± 0.06	*t* = 0.68	0.497
Visceral fat area (cm^2^), M (Q_1_, Q_3_)	117.10 (86.75, 144.95)	119.35 (87.00, 154.30)	115.40 (86.75, 132.55)	*Z* = −0.92	0.358
Blood urate level (μmol/L), M (Q_1_, Q_3_)	405.00 (349.00, 469.25)	356.00 (321.50, 387.00)	476.00 (445.00, 520.00)	*Z* = −12.02	**< 0.001**
25 (OH)D level (ng/ml), M (Q_1_, Q_3_)	22.15 (18.78, 25.90)	22.20 (18.72, 26.40)	22.00 (18.80, 25.62)	*Z* = −0.04	0.969
Vitamin D deficiency, *n* (%)				χ^2^ = 0.08	0.784
No	126 (63.00)	69 (62.16)	57 (64.04)		
Yes	74 (37.00)	42 (37.84)	32 (35.96)		
Total cholesterol (mmol/L), M (Q_1_, Q_3_)	4.59 (4.06, 5.05)	4.57 (3.96, 4.88)	4.63 (4.16, 5.13)	*Z* = −1.28	0.199
LDL cholesterol (mmol/L), M (Q_1_, Q_3_)	2.62 (2.23, 3.07)	2.54 (2.19, 2.89)	2.71 (2.38, 3.23)	*Z* = −2.05	**0.041**
HDL cholesterol (mmol/L), M (Q_1_, Q_3_)	1.16 (1.05, 1.29)	1.18 (1.09, 1.34)	1.15 (1.02, 1.27)	*Z* = −1.59	0.113
Triglyceride (mmol/L), M (Q_1_, Q_3_)	1.24 (0.91, 1.71)	1.15 (0.87, 1.54)	1.31 (0.98, 1.81)	*Z* = −1.45	0.146
Dyslipidemia, *n* (%)				χ^2^ = 9.01	**0.003**
No	122 (61.00)	78(70.27)	44 (49.44)		
Yes	78(39.00)	33(29.73)	45 (50.56)		
Fasting insulin (pmol/L), M (Q_1_, Q_3_)	183.50 (116.25, 272.25)	155.50 (109.75, 231.32)	215.00 (147.75, 299.25)	*Z* = −2.60	**0.009**
Hyperinsulinemia, *n* (%)				χ^2^ = 11.99	**< 0.001**
No	97 (48.50)	66 (59.46)	31 (34.83)		
Yes	103 (51.50)	45 (40.54)	58 (65.17)		
HbA1c (%), M (Q_1_, Q_3_)	5.80 (5.60, 6.10)	5.80 (5.60, 6.10)	5.70 (5.50, 5.93)	*Z* = −1.49	0.135
FBG (mmol/L, M (Q_1_, Q_3_)	5.00 (4.75, 5.22)	5.01 (4.74, 5.23)	4.99 (4.75, 5.19)	*Z* = −0.35	0.725
ALT (U/L), M (Q_1_, Q_3_)	25.00 (17.00, 41.00)	23.00 (16.00, 31.00)	31.00 (19.50, 55.00)	*Z* = −3.56	**< 0.001**
ALT level, *n* (%)				χ^2^ = 15.04	**< 0.001**
Normal	150 (75.00)	96 (86.49	56 (62.92)		
Abnormal	50 (25.00)	15 (13.51)	33 (37.08)		

t, *t*-test; Z, Mann-Whitney test; χ^2^, Chi-square test; SD, standard deviation; M, Median; Q_1_, 1st Quartile; Q_3_, 3rd Quartile. Bold values represent results with statistical significance (*P* < 0.05).

### 3.2 Multivariate logistic regression analysis of risk factors for HUA in adolescents with obesity

Taking the occurrence of HUA as the dependent variable (0 = non-HUA, 1 = HUA), the variables with *P* < 0.05 in univariate analysis, including Age (0 = ≤ 12 y, 1 = >12 y), BMIZ(0 = ≤ 2.7, 1 = >2.7), dyslipidemia (0 = No,1 = Yes), hyperinsulinemia (0 = no, 1 = yes) and ALT abnormal (0 = normal, 1 = abnormal), were included as independent variables in the multivariate logistic regression model. In addition, gender (male = 1, female = 2) was also incorporated into the model as a covariate despite no statistically significant difference in univariate analysis, so as to eliminate the potential confounding effect of sex on the research results.

The results showed that age > 12 years (OR = 2.60, 95%CI: 1.19–5.68, *P* = 0.017), hyperinsulinemia (OR = 2.33, 95%CI: 1.11–4.92, *P* = 0.026) and abnormal ALT (OR = 2.44, 95%CI: 1.02–5.87, *P* = 0.046) were independent correlates of HUA in adolescents with obesity after multivariable adjustment. BMIZ, dyslipidemia and gender were not significantly associated with HUA (*P* > 0.05). The detailed results are shown in Table 2.

**TABLE 2:** Multivariate logistic regression analysis of risk factors for hyperuricemia in adolescents with obesity.

Variables	β	S.E	Z	*P*	OR (95%CI)
Intercept	−1.13	0.36	−3.14	**0.002**	0.32 (0.16 ∼ 0.66)
Age (0 = ≤ 12,1 = >12)					
0					1.00 (Reference)
1	0.96	0.40	2.40	**0.017**	2.60 (1.19 ∼ 5.68)
Gender (male = 1,female = 2)					
1					1.00 (Reference)
2	−0.61	0.43	−1.41	0.157	0.54 (0.23 ∼ 1.27)
Hyperinsulinemia (0 = no,1 = yes)					
0					1.00 (Reference)
1	0.85	0.38	2.23	**0.026**	2.33 (1.11 ∼ 4.92)
Dyslipidemia(0 = no,1 = yes)					
0					1.00 (Reference)
1	0.67	0.38	1.74	0.081	1.95 (0.92 ∼ 4.13)
ALT (0 = normal, 1 = abnormal)					
0					1.00 (Reference)
1	0.89	0.45	2.00	**0.046**	2.44 (1.02 ∼ 5.87)
BMIZ (0 = < 2.7,1 = ≥ 2.7)					
0					1.00 (Reference)
1	0.11	0.38	0.29	0.769	1.12 (0.53 ∼ 2.38)

OR, odds ratio, CI, confidence interval. Bold values represent results with statistical significance (*P* < 0.05).

Two pre-specified interaction terms (age × gender and BMIZ × hyperinsulinemia) were then incorporated to test effect-modification. The interaction P-value for age-by-gender was 0.726, and the *P*-value for BMIZ-by-hyperinsulinemia was 0.534. Neither interaction achieved statistical significance. Bayesian-based generalized causal-mediation analysis with 50 simulation iterations (adjusted for age, gender and ALT) was performed to explore whether continuous fasting-insulin mediated the association between continuous BMIZ and hyperuricemia. The total effect and direct effect of BMIZ on HUA exhibited borderline-statistical-significance (β = 0.08, 95%CI: −0.01 to 0.12, *P* = 0.080; β = 0.08, 95%CI: −0.01 to 0.13, *P* = 0.080). The indirect effect transmitted through fasting insulin was close to zero (β = 0.00, 95%CI: −0.02 to 0.01, *P* = 0.840). No statistically-significant indirect pathway was detected, thus our data do not provide statistical evidence for fasting insulin as a mediating variable linking adiposity and HUA.

### 3.3 Restricted cubic spline (RCS) analysis

**FIGURE 1:**
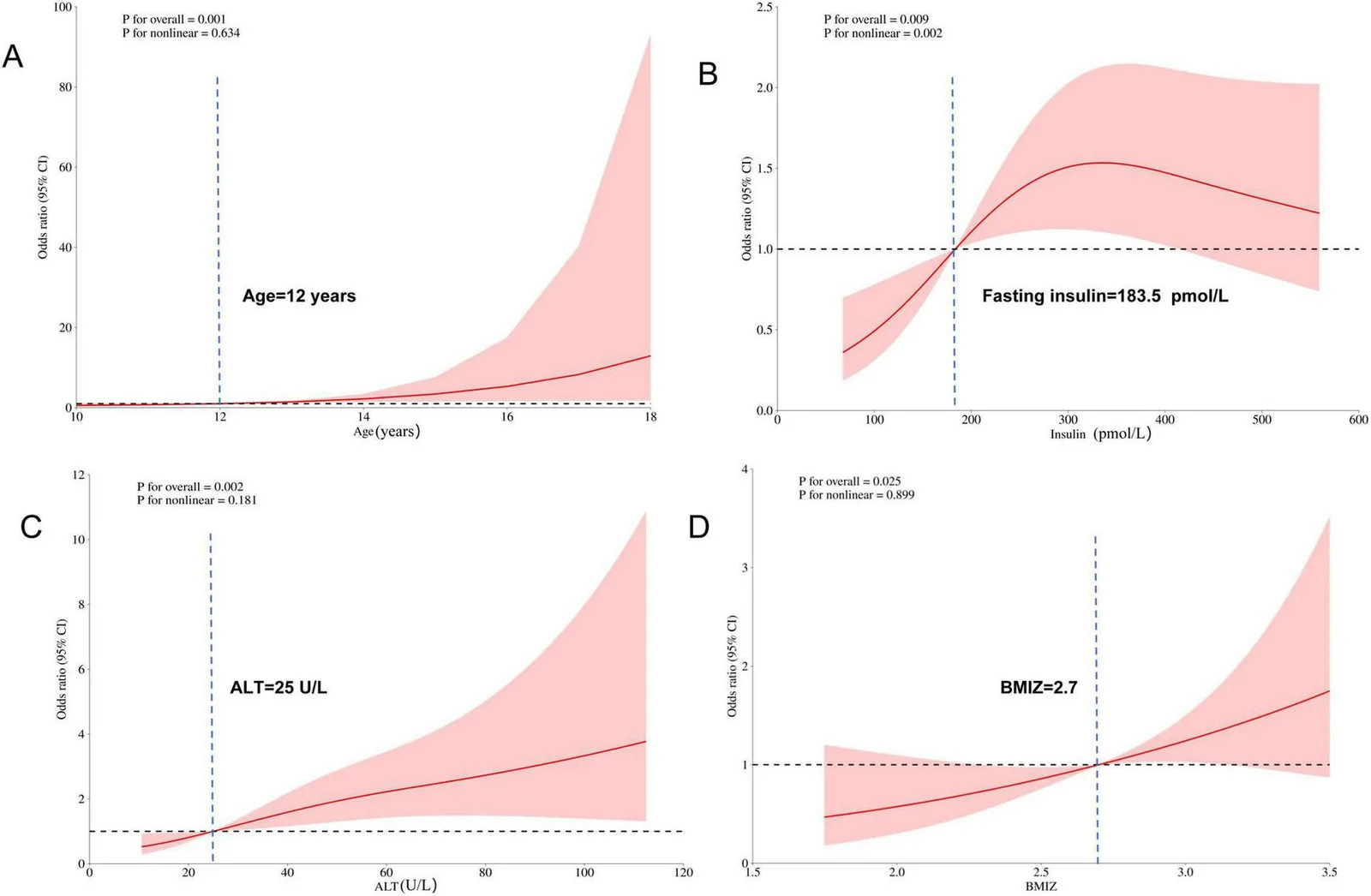
Restricted cubic spline plots for associations between continuous predictors and log odds of hyperuricemia. Three knots at the 10th, 50th, and 90th percentiles were used for each variable in unadjusted RCS models. **(A)** Age (reference = 12 y); **(B)** Fasting insulin (reference = 183.5 pmol/L); **(C)** ALT (reference = 25 U/L); **(D)** BMIZ (reference = 2.7). The solid line represents odds ratio, and shaded areas represent 95% confidence intervals.

Restricted cubic spline (RCS) models with three knots placed at the 10th, 50th, and 90th percentiles were used to characterize dose-response relationships between continuous predictors (age, fasting insulin, ALT, and BMIZ) and the log odds of HUA. The median value (50th-percentile knot) for each variable was set as the reference point where the odds ratio (OR) was fixed at 1. For age, the RCS model incorporated three knots located at 10 years (10th percentile), 12 years (50th percentile), and 15 years (90th percentile), with 12 years defined as the reference. Age showed a significant overall association with HUA risk (*P* for overall = 0.001), while the test for nonlinearity was non-significant (*P* for nonlinear = 0.634), indicating an approximately linear positive relationship; the odds of HUA increased with advancing chronological age. For fasting insulin, knots were set at 86.71 pmol/L (10th percentile), 183.5 pmol/L (50th percentile), and 431.8 pmol/L (90th percentile), and 183.5 pmol/L served as the reference. There was a significant overall association between fasting insulin and HUA risk (*P* for overall = 0.009). A statistically significant nonlinear relationship was also observed (*P* for nonlinear = 0.002), suggesting that the rate of increase in HUA odds changed across the range of fasting-insulin concentrations. For ALT, knots were located at 12 U/L (10th percentile), 25 U/L (50th percentile), and 71.7 U/L (90th percentile), with 25 U/L as the reference value. ALT exhibited a significant overall association with HUA risk (*P* for overall = 0.002), whereas the nonlinear test was not significant (*P* for nonlinear = 0.181), supporting an approximate linear positive association between ALT levels and HUA odds. For BMIZ, three knots were placed at 2.05 (10th percentile), 2.7 (50th percentile), and 3.4 (90th percentile), and 2.7 was taken as the reference. BMIZ demonstrated a significant overall association with HUA risk (*P* for overall = 0.025), with no evidence of nonlinearity (*P* for nonlinear = 0.899), showing a roughly linear trend of rising HUA odds with higher BMIZ values. The fitted RCS curves with shaded 95% confidence intervals are presented in Figure 1.

## 4 Discussion

This cross-sectional study revealed a high prevalence of HUA (44.50%, 89/200) among adolescents with obesity in Guangxi, China, with a prevalence of 47.59% (69/145) in males and 36.36% (20/55) in females. Multivariate logistic regression identified older age (> 12 years), hyperinsulinemia, and abnormal ALT as independent factors associated with HUA. Restricted cubic spline (RCS) analyses further demonstrated significant dose-response relationships between age, fasting insulin, ALT, BMIZ and HUA risk, reinforcing the robustness of these associations. These findings underscore the importance of comprehensive metabolic and hepatic assessments in the clinical management of adolescents with obesity. Of note, given the cross-sectional design of this investigation, all reported associations are correlational; temporal ordering and causal relationships between these metabolic phenotypes cannot be inferred from the present dataset.

The prevalence of HUA observed in our study (44.50%) is substantially higher than that reported in general pediatric populations in China (23.3%), highlighting the particular vulnerability of adolescents with obesity to elevated uric acid levels (12). Notably, this prevalence also exceeds the 30.6% HUA prevalence reported among obese adults in Guangxi using the same SUA cut-off (≥ 420 μmol/L) (13), suggesting that adolescents with obesity may face an even higher metabolic burden than their adult counterparts. This elevated prevalence aligns with findings from other studies in obese youth, with reported rates ranging from 50.6 to 64.5% depending on obesity severity and regional characteristics (17). However, the prevalence in the present study was slightly lower than previously reported rates of HUA in adolescents with obesity, which may be attributed to the fact that most previous studies adopted different diagnostic criteria, defining HUA as > 420 μmol/L in males and > 360 μmol/L in females (17). The elevated HUA burden may be attributed to the metabolic consequences of excess adiposity, including increased purine turnover, enhanced uric acid production from fructose metabolism, and insulin resistance-mediated reduction in renal uric acid excretion (9). In our cohort, body fat percentage and visceral fat area showed no significant differences between HUA and non-HUA groups, implying that overall adiposity severity reflected by BMIZ, rather than differential fat distribution, appears to correlate more strongly with HUA risk in this clinical sample.

Our multivariable analysis identified hyperinsulinemia as an independent factor associated with HUA (OR = 2.33). Insulin-resistance-related compensatory hyperinsulinemia may increase renal urate reabsorption by modulating renal urate transporters and accelerate hepatic ATP degradation to promote uric acid overproduction (18, 19). Existing Mendelian randomization analyses support directional associations from insulin resistance toward higher serum uric acid, though bidirectional metabolic feedback cannot be fully excluded in cross-sectional cohorts (20, 21). Our RCS analysis additionally identified a significant nonlinear dose-response relationship between fasting insulin and HUA risk, indicating that the rate of HUA risk elevation changes across the spectrum of fasting insulin concentrations in obese adolescents. These findings reinforce the close linkage between hyperinsulinemia and hyperuricemia among this high-risk pediatric population.

Another key finding of the present study is that abnormal ALT emerged as an independent risk factor for HUA (OR = 2.44) in adolescents with obesity. The RCS analysis also revealed a significant overall positive dose-response relationship between ALT and HUA risk. Elevated ALT, a marker of hepatocellular injury, is commonly observed in adolescents with obesity with non-alcoholic fatty liver disease (NAFLD) (22). The liver plays a central role in purine metabolism, and hepatic insulin resistance may promote *de novo* lipogenesis and increased uric acid generation (23). Experimental evidence indicates bidirectional interactions: elevated uric acid may trigger hepatic NLRP3 inflammasome activation and oxidative stress to worsen hepatocyte injury; conversely, liver metabolic dysfunction may further disturb intra-hepatic purine turnover and raise serum urate (24). A recent population-based study among US youth aged 13–20 years also identified elevated ALT as an independent risk factor for HUA, further corroborating the association between hepatic injury and hyperuricemia in young populations (25). Nevertheless, because of the cross-sectional nature of our study, we cannot determine which abnormality occurs first in individual patients. Our results add real-world clinical evidence supporting the co-occurrence of hepatic injury and hyperuricemia among obese adolescents, highlighting the clinical value of routine liver-function monitoring.

Older age (> 12 years) was independently associated with higher HUA risk (OR = 2.60), consistent with previous pediatric reports and national meta-analytic evidence demonstrating elevated HUA risk starting from 12 years of age (17, 26, 27). Epidemiological data show that HUA prevalence remains low before age 12 and rises substantially in mid-adolescence, with growing gender-related disparity after puberty (26). Physiologically, rapid pubertal muscle growth accelerates purine turnover and urate production (28). Increased androgen levels suppress renal urate excretion in boys; although estrogen confers partial protective effects on urate metabolism in girls, this effect cannot fully counteract elevated urate synthesis (29). Unfavorable lifestyle habits such as reduced physical activity and high-sugar food intake may further exacerbate metabolic risk in older adolescents. Our RCS model demonstrated an approximately linear positive dose-response between age and HUA risk, supporting enhanced metabolic screening for obese adolescents entering mid-adolescence.

Although dyslipidemia was significantly associated with HUA in univariate analysis, it lost statistical significance in multivariate logistic regression, indicating that dyslipidemia may not independently contribute to the risk of HUA in obese adolescents, and its association with HUA may be mediated by other metabolic factors such as insulin resistance and hepatic injury.

Several limitations should be acknowledged. First, the cross-sectional design precludes causal inferences; prospective longitudinal studies are needed to establish temporality. Second, this single-center clinical sample from a specialized weight-management clinic may introduce referral bias and limit generalizability, although the male predominance reflects regional obesity epidemiology in Guangxi. Third, renal function biomarkers (creatinine, eGFR) were not uniformly available for all participants, limiting adjustment for renal confounding. Fourth, standardized Tanner staging was not collected, precluding full adjustment for pubertal hormonal effects. Fifth, quantitative dietary and physical activity data were incomplete and could not be formally incorporated into analyses. Sixth, the absence of longitudinal follow-up prevented assessment of intervention effects on SUA trajectories and clinical outcomes. In future research, we will adopt more rigorous dietary questionnaires and standardized investigation methods to collect detailed information on lifestyle factors including dietary intake and physical activity, and conduct long-term follow-up of the study population to observe the clinical effects of targeted nutritional interventions on improving HUA and related metabolic abnormalities in adolescents with obesity.

Despite these limitations, our study has important clinical and public health implications. The high prevalence of HUA among adolescents with obesity, exceeding that of obese adults in the same region, necessitates routine screening for uric acid levels in pediatric weight management clinics in Guangxi and other southern Chinese regions. The identification of hyperinsulinemia and hepatic injury as independent risk factors suggests that clinical nutrition interventions should extend beyond weight reduction to include improvement of insulin sensitivity and protection of hepatic function, with particular attention to older adolescents. Strategies such as reducing refined carbohydrate and fructose intake, increasing dietary fiber, and promoting regular physical activity may concurrently address obesity, insulin resistance, and HUA (30). However, it is important to note that current international guidelines do not universally recommend pharmacological treatment for asymptomatic HUA in adolescents, and the cost-effectiveness of routine screening warrants further investigation. Our findings primarily support screening as a tool for identifying high-risk individuals who may benefit from intensive lifestyle interventions, rather than as a justification for pharmacotherapy. Additionally, our findings provide valuable baseline data for the implementation of China’s “Action Plan of Five-Health Promotion for Children and Adolescents (2026–2030),” supporting the development of region-specific metabolic management strategies for adolescents with obesity.

## 5 Conclusion

In conclusion, this study demonstrates a high prevalence of HUA (44.50%) among adolescents with obesity in Guangxi, China, exceeding the reported 30.6% HUA prevalence among obese adults from the same region. Older age (> 12 years), hyperinsulinemia, and elevated ALT are independently associated with HUA risk in this clinical cohort. These findings highlight the necessity for comprehensive metabolic assessments including uric acid, fasting insulin, and liver-function markers for obese adolescents, with enhanced attention for patients older than 12 years. Targeted clinical-nutrition interventions aiming to improve insulin sensitivity and protect hepatic function may mitigate HUA-related metabolic risk. Further large-scale multi-center prospective cohort investigations are needed to clarify causal biological pathways, and intervention trials are required to evaluate the long-term effectiveness of integrated metabolic-management strategies for adolescent HUA and its chronic sequelae.

## Data Availability

The original contributions presented in this study are included in this article/supplementary material, further inquiries can be directed to the corresponding authors.
